# The Partial Reconstruction Symplectic Geometry Mode Decomposition and Its Application in Rolling Bearing Fault Diagnosis

**DOI:** 10.3390/s23177335

**Published:** 2023-08-22

**Authors:** Yanfei Liu, Junsheng Cheng, Yu Yang, Guangfu Bin, Yiping Shen, Yanfeng Peng

**Affiliations:** 1 State Key Laboratory of Advanced Design and Manufacturing for Vehicle Body, Hunan University, Changsha 410082, China; hndxlyf@hnu.edu.cn (Y.L.); zjsignal@hnu.edu.cn (Y.Y.); 2 Hunan Provincial Key Laboratory of Health Maintenance for Mechanical Equipment, Hunan University of Science and Technology, Xiangtan 411201, China; 21010301016@mail.hnust.edu.cn (G.B.); 22010302009@mail.hnust.edu.cn (Y.S.); 22020301060@mail.hnust.edu.cn (Y.P.)

**Keywords:** PRSGMD, RCMFE, SGMD, rolling bearings, fault diagnosis

## Abstract

Extracting the fault characteristic information of rolling bearings from intense noise disturbance has been a heated research issue. Symplectic geometry mode decomposition (SGMD) has already been adopted for bearing fault diagnosis due to its advantages of no subjective customization of parameters and the ability to reconstruct existing modes. However, SGMD suffers from rapidly decreasing calculation efficiency as the amount of data increases, in addition to invalid symplectic geometry components affecting decomposition accuracy. The regularized composite multiscale fuzzy entropy (RCMFE) operator is constructed to evaluate the complexity of each initial single component and minimize the residual energy. Combined with the partial reconstruction threshold indicator to filter out specific significant initial single components, the raw signal can be decomposed into multiple physically meaningful symplectic geometric mode components. Therefore, the decomposition efficiency and accuracy can be enhanced. Thus, a rolling bearing fault diagnosis method is proposed based on partial reconstruction symplectic geometry mode decomposition (PRSGMD). Both simulated and experimental analysis results show that PRSGMD can improve the speed of SGMD analysis while increasing the decomposition accuracy, thereby augmenting the robustness and effectiveness of the algorithm.

## 1. Introduction

Rolling bearings are critical elements of mechanical equipment systems, the failure of which always causes a chain reaction, resulting in the occurrence of machine damage of varying degrees that may lead to collapses or even accidents in severe cases. Therefore, the health status of rolling bearings is directly related to the reliability of mechanical equipment operation [1,2,3]. Therefore, to guarantee their security and stability during operation, rolling bearing real-time service state monitoring and fault diagnosis is essential [4,5]. Yet, due to the complicated internal composition of the equipment and the harsh operating environment, the captured vibration signals are often coupled with multiple component vibratory modes and noise. Hence, the key to accurately diagnosing bearing faults is extracting fault characteristic information from the vibration signal with disturbance information [6,7]. Traditional methods for fault feature information extraction include Fourier-transform-based spectral analysis methods, short-time Fourier transform (STFT), Wigner distribution (WD), and wavelet transform (WT) [8,9,10,11]. Due to the use of fixed basis functions, these analysis methods often lead to analysis results that lose physical significance and fail to extract the intrinsic characteristics of the signal [12,13]. Many experts and scholars from home and abroad have carried out much research in order to select the basis function or its parameters automatically according to the characteristics of the signal itself during signal decomposition so that the intrinsic characteristics of the mechanical fault vibration signal can be extracted effectively, and many beneficial results have been published. According to how the basic functions are identified, there are two major categories of adaptive signal decomposition methods: parametric and non-parametric.

Among them, the parametric adaptive signal decomposition method needs to select the basis function according to the characteristics of the signal in advance, then determine the optimal parameters or coefficients of the basis function adaptively to realize the optimal matched signal during decomposition [14,15], whereas the non-parametric adaptive signal decomposition method automatically selects the basis function according to the characteristics of the signal itself, the basis function of which does not have a definite analytic expression [16,17].

The non-parametric adaptive signal analysis method can automatically choose the basis function or its parameters according to the characteristics of the signal itself in the process of signal decomposition in order to obtain physically meaningful components. It thus can effectively extract the intrinsic characteristics of mechanical fault vibration signals. Compared with the parametric adaptive signal analysis method, the nonparametric adaptive signal analysis method does not need to construct a complete dictionary library based on the characteristics of the signal in advance, resulting in higher adaptiveness as well as decomposed components with physical significance. This is why it has been popularly adopted for mechanical fault diagnosis. At present, the common non-parametric adaptive signal analysis methods include Hilbert–Huang transform (HHT) [18], local mean decomposition (LMD) [19] and local characteristic-scale decomposition (LCD) [20], etc. The ideas of these methods are similar: obtaining physically meaningful decomposition results by fitting the extreme value points. Compared with the parametric adaptive signal analysis methods, these methods are superior in terms of adaptiveness and physical significance of decomposition results, but they have two drawbacks in common. Firstly, they must all fit the extreme value points in the decomposition process. At the same time, such problems as over-enveloping, under-enveloping, frequency confusion, endpoint effect, etc., occur inevitably in the process of fitting the extreme value points. Next, there is no rigorous mathematical justification for whether the defined single-component signals are physically meaningful or not.

Combining with the symplectic geometry theory, Pan et al. proposed the symplectic geometry mode decomposition (SGMD) method [21], and by decomposing the signal, certain symplectic geometry components (SGCs) with independent modes can be obtained. The Hamiltonian matrix’s eigenvalues are solved utilizing the symplectic geometric similar transform via SGMD, which is then used to reconstruct the single-component signal with the corresponding eigenvectors. The SGMD possesses advantages in that there is no subjective customization of parameters, and it can effectively be used to reconstruct existing modes and eliminate noise. However, SGMD suffers from the defects that the calculation efficiency decreases rapidly as the amount of data increases, in addition to invalid SGCs affecting the decomposition accuracy during reconstruction. Aiming at these issues, this paper takes advantage of the fact that composite multiscale fuzzy entropy (CMFE) can effectively evaluate the complexity of each initial single component of SGMD, as well as being able to overcome mutations in the signal initial single component similarity index [22]. Firstly, the RCMFE operator is constructed to evaluate the complexity of each initial single component after reconstruction and constrain the residual energy to be minimized; then, it is combined with the constructed partial reconstruction threshold indicator to terminate the merge. Accordingly, this paper proposes a signal-denoising method based on partial reconstruction SGMD. Relative to the original version, PRSGMD only needs to deal with the part of the initial single component that contains significant modes, and the computation efficiency does not decrease with the increase in data amount, which can effectively improve decomposition speed. Meanwhile, PRSGMD can increase the decomposition accuracy while improving the speed of SGMD analysis owing to removing the effects of noisy and other invalid modes on decomposition results. The analysis results show that PRSGMD is more effective than the existing adaptive signal decomposition algorithms in denoising and extracting fault characteristic information.

The remaining parts are arranged in the following manner. Section 2 suggests the PRSGMD approach, which is motivated by the fundamental SGMD theory; Section 3 compares the PRSGMD, SGMD, VMD, and EEMD in simulation; in Section 4, the experimental signals are analyzed adopting the PRSGMD method; the last section is the conclusion.

## 2. The Theory of the PRSGMD

### 2.1. SGMD

SGMD solves the Hamiltonian matrix eigenvalues by adopting a symplectic geometric similar transform and reconstructs SGCs based on their relevant eigenvectors, thereby denoising the complex signal and performing the adaptive decomposition. SGMD consists of the following three major procedures.

1. Phase space reconstruction

Set the original signal time sequence as ${x=x}_{1}{,\;x}_{2},\ldots {,\;x}_{n}$, where n is the data length. From the taken embedding theorem, employing a time sequence deferred topology equivalence on a one-dimensional signal can reconstruct a poly-dimensional signal and thus obtain the trajectory matrix X, where d is the embedding dimension, τ. is the delay time, and m=n−(d−1)τ.

(1) $$\;X=\left(\begin{array}{c} x_{1}\\ \vdots \\ x_{m} \end{array}\begin{array}{ccc} x_{1+\tau } & \ldots & x_{1+(d-1)\tau }\\ \vdots & \ddots & \vdots \\ x_{m+\tau } & \cdots & x_{m+(d-1)\tau } \end{array}\right)$$

2. Symplectic Obtain s geometric initial single component

In order to construct the Hamiltonian matrix, the autocorrelation analysis is carried out on the trajectory matrix to obtain the covariance symmetry matrix A:

(2) $${A=X}^{T}X$$

Decomposition of the matrix $A^{2}$ yields the eigenvector matrix Q, where $Q_{i}(i=1,\;2,\ldots ,\;d)$ is the eigenvector of the matrix A corresponding to the eigenvalue $\sigma_{i}$.

The transformed coefficient matrix $S_{i}{\;=Q}_{i}{}^{T}X^{T}$ is gained by the unitary matrix eigenvectors and the trajectory matrix, and then it is converted to gain the initial single component matrix Z.

(3) $$Z_{i}{\;=Q}_{i}S_{i}$$

Diagonal averaging is employed to convert the initial single component matrix Z to obtain the symplectic geometric initial single component $Y_{i}{\;=[y}_{1}{,\;y}_{2},\ldots {,\;y}_{k},\ldots {,\;y}_{n}]$, where i=[1, 2,…, d].

(4) $$y_{k}=\left\{\begin{array}{l} \frac{1}{k}\overset{k}{\underset{p=1}{\sum }}z_{p,\;k-p+1}^{*}\;\;1\leq k\leq d^{*}\\ \frac{1}{d^{*}}\overset{d^{*}}{\underset{p=1}{\sum }}z_{p,\;k-p+1}^{*}\;{\;d}^{*}\leq k\leq m^{*}\\ \frac{1}{n-k+1}\overset{{n-m}^{*}+1}{\underset{p=k-m^{*}+1}{\sum }}z_{p,\;k-p+1}^{*}{\;m}^{*}\leq k\leq n \end{array}\right.$$

3. Single Component Reconstruction

d single-component signals are acquired through the trajectory matrix decomposition, but at this time, not all single components are independent of each other, so each group of components is likely to possess identical cyclic components, identical frequency components, etc. As a result, each initial single component needs to be recomposed. SGMD utilizes period similarity as the evaluation index; firstly, the matrix Y is a d × n matrix, as the main parts are arranged in its front row, so the period similarity is compared between $Y_{1}$ and the remaining components. The first component ${\mathrm{SGC}}_{1}$ is acquired from the reconstruction of components with high similarity, while those who have been involved in the reconstruction of ${\mathrm{SGC}}_{1}$ will not participate in the rest of the reconstruction process; the remainder is denoted as $G^{1}$, then the remainder signal $g_{1}$ is produced by summarizing the remainder component matrix to calculate the NMSE (normalized mean squared error) between the remainder signal and the raw signal, when. W it is smaller than the specified threshold, decomposition stops; if not, the remainder component matrix is treated as the original matrix to continue iteration before reaching the iteration termination criteria. Set N as the number of component sequences obtained; then, the final decomposition result is

(5) $$\;x(n)=\overset{N}{\underset{h=1}{\sum }}{\mathrm{SGC}}^{h}\left(n\right)+g^{\left(N+1\right)}\left(n\right)$$

### 2.2. Composite Multiscale Fuzzy Entropy

Given that different characteristic information and noise of the signal tend to be distributed at different scales, the initial single component containing noise tends to be of higher complexity, while the initial single component containing fault characteristic information is of inferior complexity. Therefore, the complexity of each initial single component is evaluated and ordered using the CMFE.

CMFE utilizes fuzzy entropy to overcome the mutation of the similarity index of the SGC component in the signal. Meanwhile, aiming at the effect on fuzzy entropy calculation due to the shortening of the time sequence in the coarse granulation process, the mean value of the fuzzy entropy of different crude granulation sequences under the same scale factor is used as the fuzzy entropy value under this scale factor. CMFE is calculated as follows:

At first, for a given signal *x*(*n*) with N data points, calculate different coarse-grained time sequences $y_{k}{}^{\left(\tau \right)}{=\{y}_{k,1}{}^{\left(\tau \right)}{,y}_{k,2}{}^{\left(\tau \right)},K{,y}_{k,j}{}^{\left(\tau \right)}\}$ with scale factor τ, where

(6) $$y_{k,j}{}^{\left(\tau \right)}=\frac{1}{\tau }\overset{j\tau +k-1}{\underset{i=(j-1)\tau +k}{\sum }}x_{i}$$

where $1\;\leq \;j\;\leq \;\frac{N}{\tau }$, 1 ≤ k ≤ τ.

Next, for each scale factor, the fuzzy entropy of each coarse-grained sequence y(τ)k(1 ≤ k ≤ τ) is calculated, then the mean value of the τ entropy values is calculated, making FE(⋅) the fuzzy entropy calculation of the signal; thus, the CMFE of this scale factor τ is obtained:

(7) $$\mathrm{CMFE}[x(n)]=\frac{1}{\tau }\overset{\tau }{\underset{k=1}{\sum }}{\mathrm{FE}(y}_{\tau }{}^{\left(k\right)},m,n,r)$$

### 2.3. Partial Reconstruction Symplectic Geometry Mode Decomposition

**Figure 1:**
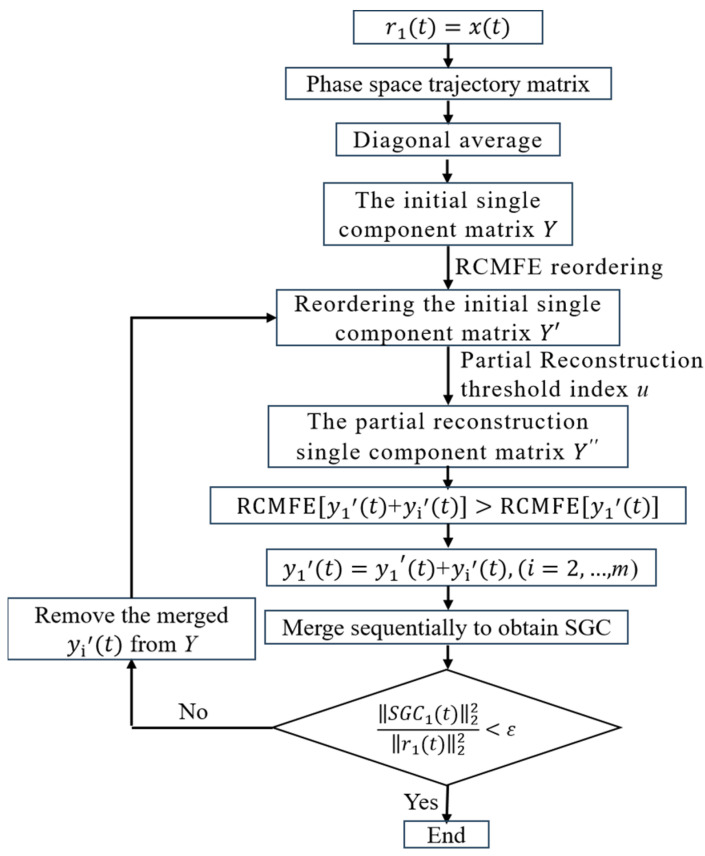
The flow chart of PRSGMD.

After transforming the initial single component matrix Z by diagonal averaging, SGMD obtains d initial symplectic geometric single components $Y_{i}$, where d is the embedding dimension, usually set as n/3. Therefore, when SGMD is used to process signals with data length n, the single component reconstruction link needs to carry out cyclic iteration on n/3 initial single components $Y_{i}$ to compare the similarity between $Y_{i}$ and other initial single components. When the amount of data increases, the calculation amount of SGMD increases rapidly correspondingly, and the calculation time becomes longer, which is not conducive to the practicability and effectiveness of SGMD. At the same time, the initial single component containing invalid modes, such as noise, is not distinguished during reconstruction, which affects the decomposition accuracy of SGC. Aiming at the above deficiencies, the Partial Reconstruction Symplectic Geometry Modeprsgm method is proposed in this paper. For the signal $x\left(t\right)$, the iterative process of the PRSGMD method is as follows, and the iterative flow is shown in Figure 1.

(1) Set $r_{1}\left(t\right)=x\left(t\right)$.

(2) Construct the phase space trajectory matrix X.

(8) $$\;X=\left(\begin{array}{c} r_{1}\left(1\right)\\ \vdots \\ r_{1}\left(m\right) \end{array}\begin{array}{ccc} r_{1}\left(1+\tau \right) & \ldots & r_{1}[1+(d-1)\tau ]\\ \vdots & \ddots & \vdots \\ r_{1}\left(m+\tau \right) & \cdots & r_{1}[m+(d-1)\tau ] \end{array}\right)$$

Here, n is the data length, d is the embedding dimension usually set to n/3, and τ is the delay time, m=n−(d−1)τ. Selecting the appropriate embedding dimension d and delay time τ, the corresponding reconstruction matrix X can be obtained.

(3) Obtain the initial single component matrix ${Y=[y}_{1}\left(t\right){,y}_{2}\left(t\right),\ldots {,y}_{k}\left(t\right),\ldots {,y}_{n}\left(t)\right]$ after diagonal averaging, including $y_{k}$, as shown in Equation (4).

(4) Calculate the RCMFE operator of each initial single component.

(9) $${\mathrm{RCMFE}[y}_{k}{(t)]=\|\mathrm{CMFE}[y}_{k}{\left(t)]\right\|}_{2}^{2}{+\|y}_{k}{\left(t\right)\|}_{2}^{2}$$

Among them, the scale factor τ of the CMFE is usually set to 3, and ${\|\mathrm{CMFE}[y}_{k}{\left(t)]\right\|}_{2}^{2}$ can be used to assess the complexity of the initial single component of different scales. The different characteristic information and noise of the signal tend to be distributed in different scales; the initial single component containing noise tends to be of higher complexity, while the initial single component containing fault characteristic information is of inferior complexity. $\|y_{k}{\left(t)\right\|}_{2}^{2}$ can be used to assess the initial single component energy; the larger $\|y_{k}{\left(t)\right\|}_{2}^{2}$ is, the larger the initial single component energy, and the smaller the decomposition residual.

The initial single component matrix $Y_{i}$ is reordered by RCMFE, which separates the fault characteristic information from the noise using complexity quantization under the condition that the components obtained from the decomposition are valid. Therefore, Y is sorted by the RCMFE values of $y_{k}\left(t\right)$ from largest to smallest as follows:

(10) $$Y^{\prime }{{=[y}_{1}}^{\prime }\left(t\right){{,y}_{2}}^{\prime }\left(t\right),\ldots {{,y}_{k}}^{\prime }\left(t\right),\ldots {{,y}_{n}}^{\prime }\left(t)\right]$$

(5) Build ${{u=\mathrm{RCMFE}(y}_{k}}^{\prime }{(t))/\mathrm{RCMFE}(r}_{1}\left(t)\right)$ as the partial reconstruction threshold index, selecting u > 0.001 part of the initial single component refactoring, and obtain the partial reconstruction initial single component matrix $Y^{\prime \prime }{{=[y}_{1}}^{\prime }\left(t\right){{,y}_{2}}^{\prime }\left(t\right),\ldots {{,y}_{m}}^{\prime }\left(t)\right]$ that contains significant modes of the raw signal, the. T remaining large number of weak invalid components will not participate in the reconstruction process, thus reducing the amount of calculation and improving decomposition speed.

(6) Merge ${y_{1}}^{\prime }\left(t\right)$ with the other initial single components in turn, recalculate RCMFE, merge again if it increases, and remove the merged initial single components in $Y^{\prime \prime }$ so that ${SGC}_{1}\left(t\right)$ is the final merged obtained ${y_{1}}^{\prime }\left(t\right)$.

(7) Calculate the iteration termination criterion $\|{SGC}_{1}{\left(t)\right\|}_{2}^{2}{/\|r}_{1}{\left(t)\right\|}_{2}^{2\;}\leq \;\varepsilon$, the. T value of *ε* is usually 0.001; if the termination criterion is not satisfied, go back to step (6) and obtain ${SGC}_{i}\left(t\right)$, if. I the criterion is satisfied, terminate the iteration and accomplish the whole decomposition process.

## 3. Simulation Analysis

**Figure 2:**
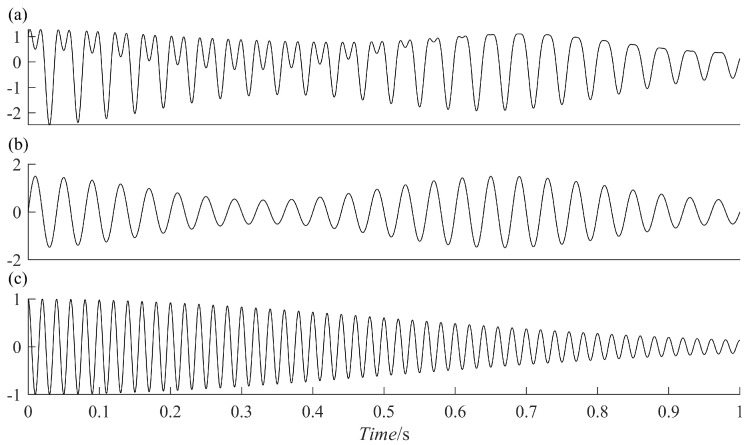
The time-domain waveforms of the simulation signal and its components. (**a**) x(t); (**b**) Thet AM–FM signal; (**c**) the vibration attenuation signal.

**Figure 3:**
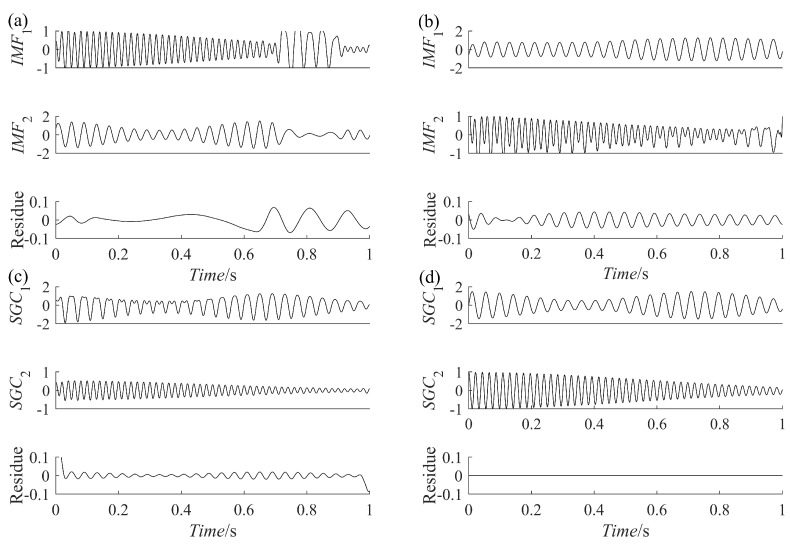
The decomposition results of the simulation signal. (**a**) The EEMD decomposition result; (**b**) Thet VMD decomposition result; (**c**) Thet SGMD decomposition result; (**d**) Thet PRSGMD decomposition result.

The PRSGMD is more accurate in signal decomposition compared to other methods. To compare and analyze, the SGMD, Variational Mode Decompositionvmd (VMD), and Ensemble Empirical Mode Decompositioneemd (EEMD) were used. First, construct the following simulated signal x(t)

(11) $$\left\{\begin{array}{l} {x(t)=x}_{1}{(t)+x}_{2}(t)\;\\ x_{1}(t)=[1+0.5\cos(10\pi t)]\sin(150\pi t)\;\\ x_{2}{(t)=\cos(100\pi t)e}^{-{2t}^{2}} \end{array}\right.$$

where x(t) consists of an AM-FM signal $x_{1}\left(t\right)$ and a vibration attenuation signal $x_{2}\left(t\right)$, Figure 2a–c represents x(t),$\;x_{1}\left(t\right)$, and $x_{2}\left(t\right)$, respectively. Use the four methods to decompose the simulated signal and compare the similarity between the decomposed components and $x_{1}\left(t\right)$ and $x_{2}\left(t\right)$ to verify the excellence of the PRSGMD. Figure 3a–d illustrates the components and the residue decomposed by the four methods. In Figure 3a, the mode mixing problem of ${IMF}_{1}$ is visually evident, indicating that the EEMD does not perform well in decomposing this signal. Figure 3b,c show the VMD and SGMD, respectively, both of which have better results compared to the EEMD. However, when comparing the amplitudes with $x_{1}\left(t\right)$ and $x_{2}\left(t\right)$, both ${SGC}_{2}$ and ${IMF}_{1}$ lose some information. Compared to the previous three methods, the SGC of PRSGMD closely matches $x_{1}\left(t\right)$ and $x_{2}\left(t\right)$ of x(t).

**Figure 4:**
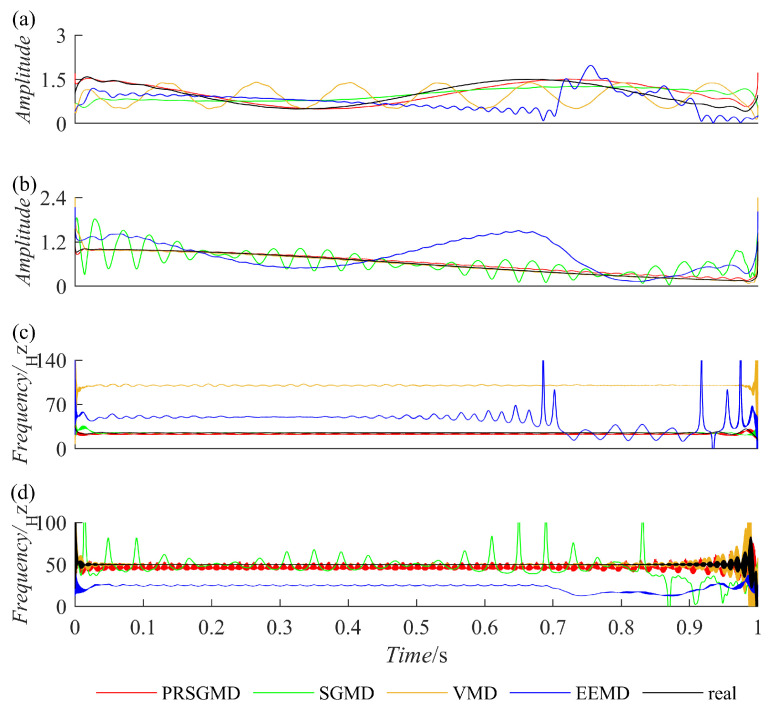
The IAs and IFs of the first two components obtained from the decomposition of the simulation signal. (**a**) The IAs of ${SGC}_{1}$ and ${IMF}_{1}$; (**b**) the IAs of ${SGC}_{2}$ and ${IMF}_{2}$; (**c**) the IFs of ${SGC}_{1}$ and ${IMF}_{1}$; (**d**) the IFs of ${SGC}_{2}$ and ${IMF}_{2}$.

**Figure 5:**
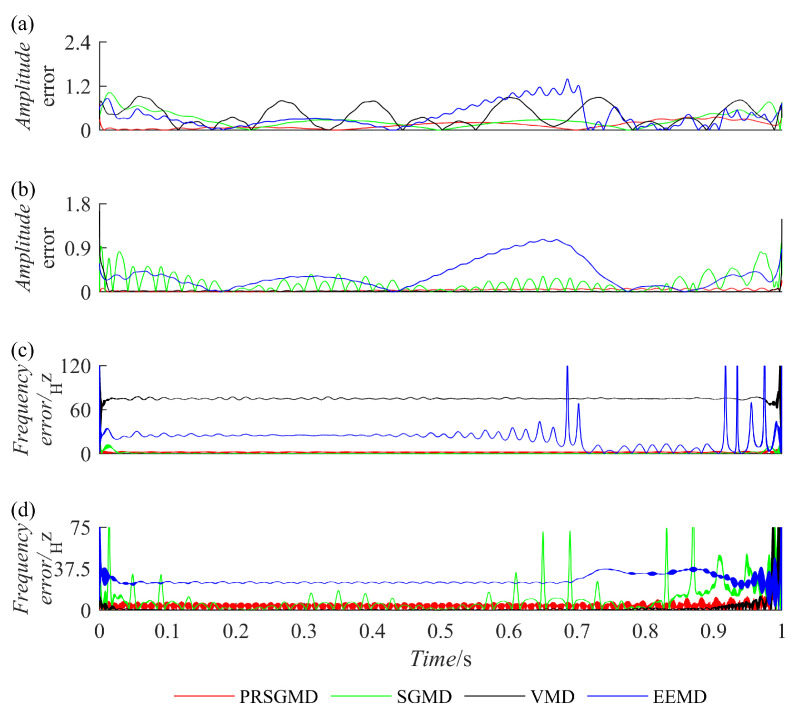
The IA errors and IF errors. (**a**) The IA errors of ${SGC}_{1}$ and ${IMF}_{1}$; (**b**) the IA errors of ${SGC}_{2}$ and ${IMF}_{2}$; (**c**) the IF errors of ${SGC}_{1}$ and ${IMF}_{1}$; (**d**) the IF errors of ${SGC}_{2}$ and ${IMF}_{2}$.

A Hilbert transform was applied to IMF and SGC to obtain their instantaneous characteristics, namely, Instantaneous Amplitudeia (IA) and Instantaneous Frequencyif (IF). They were then compared with the IA and IF of $x_{1}\left(t\right)$ and $x_{2}\left(t\right)$, resulting in Figure 4. After comparison, the absolute difference between the IA and IF of $x_{1}\left(t\right)$ and $x_{2}\left(t\right)$ was taken, resulting in Figure 5, which represents the IA errors and IF errors. Based on Figure 4 and Figure 5, it can be observed that the IA and IF of the IMF component obtained by the EEMD exhibit significant deviations from the actual values, indicating severe mode mixing. Although there are fluctuations in the IA and IF of the components decomposed by the VMD and SGMD compared to the actual values, they outperform the EEMD. Meanwhile, the SGC component obtained by the PRSGMD exhibits more accurate instantaneous amplitude and frequency, with smaller fluctuations and proximity to the actual values. It can be inferred that the component obtained by PRSGMD decomposition has higher accuracy, and the PRSGMD has a better decomposition ability.

Finally, the comprehensive performance of the four methods was further compared using metrics such as energy error ($E_{i}$), correlation coefficient ($r_{i}$), orthogonality index (IO), and computation time (T). Table 1 was created to summarize the results and compare the overall performance of the four methods. According to the comprehensive analysis in Table 1, the components obtained by the PRSGMD exhibit higher correlation coefficients and smaller energy errors with the actual values, indicating closer proximity to the actual values. Additionally, the orthogonality index of the PRSGMD decomposition result is significantly lower than that of the SGMD, indicating good orthogonality of the PRSGMD method. It is worth noting that while the decomposition speed of the PRSGMD is better than the SGMD, it is still lower than that of the EEMD and VMD.

**Figure 6:**
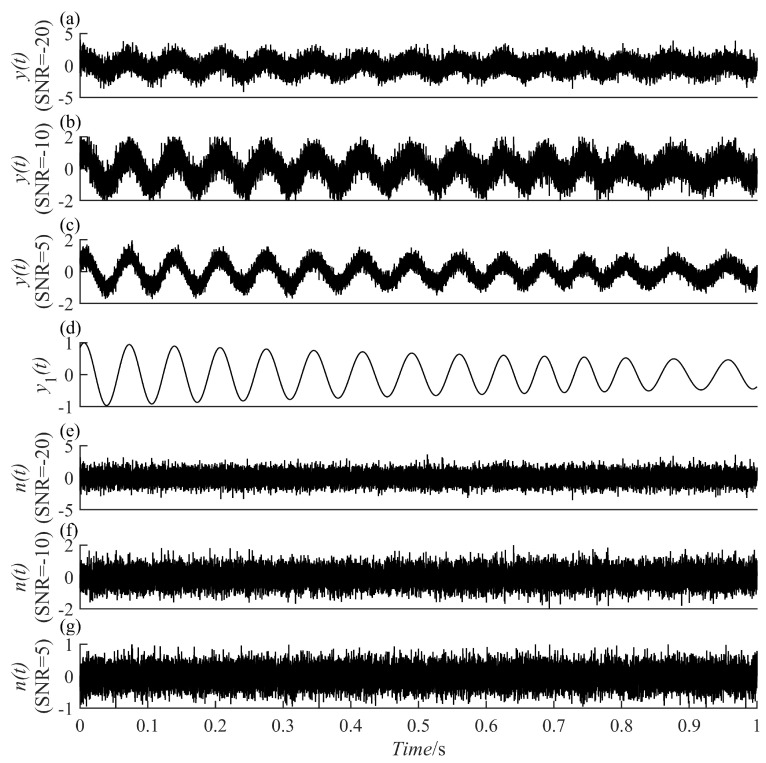
The time-domain waveforms of the simulation signal y(t) in Equation (12) and its components. (**a**–**c**) The mixed signals at SNR = 5, −10, −20, respectively; (**d**) the vibration attenuation signal; (**e**–**g**) the noise component signals in (**a**–**c**), respectively.

To validate the proposed method’s resistance to noise, a signal presented in Equation (12), y(t), was designed that consists of two parts, namely, the vibration attenuation signal and Gaussian white noise signal generated during the simulation of actual faults. The simulation signal and its component time domain waveform are depicted in Figure 6. The signal-to-noise ratios of the Gaussian white noise signal are 5dB, −10 dB–10dB, and −20 dB, respectively. Further, PRSGMD, SGMD, VMD, and EEMD were decomposed for y(t).

(12) $$\left\{\begin{array}{l} y\left(t\right){=y}_{1}\left(t\right)+n\left(t\right)\\ y_{1}\left(t\right){=e}^{-0.8t}{\sin[30\pi t+\cos(3\pi t}^{2})] \end{array}\right.$$

**Figure 7:**
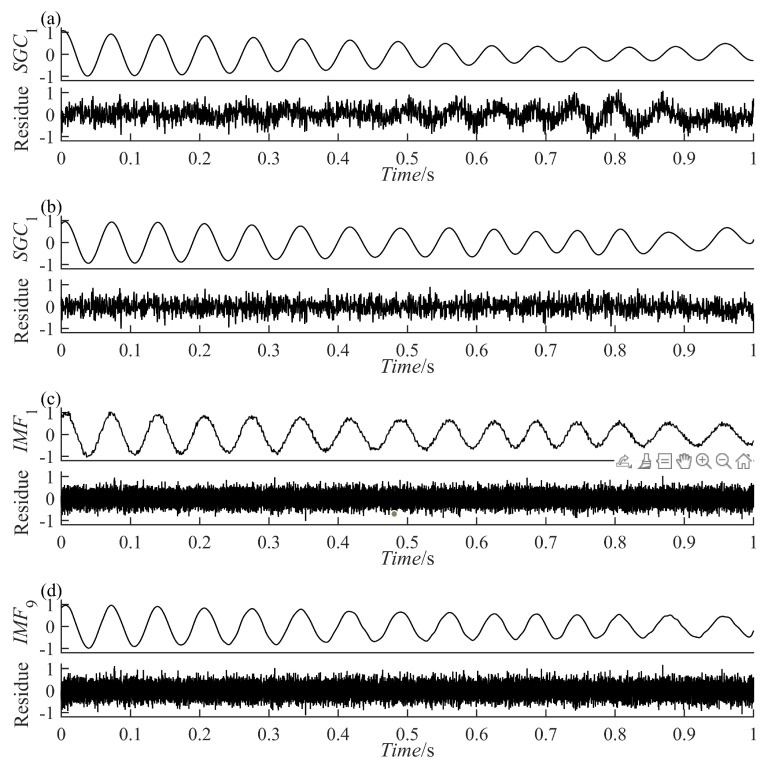
The decomposition results of the simulation signal y(t) at SNR = 5. (**a**) The PRSGMD decomposition result; (**b**) the SGMD decomposition result; (**c**) the VMD decomposition result; (**d**) The the EEMD decomposition result.

**Figure 8:**
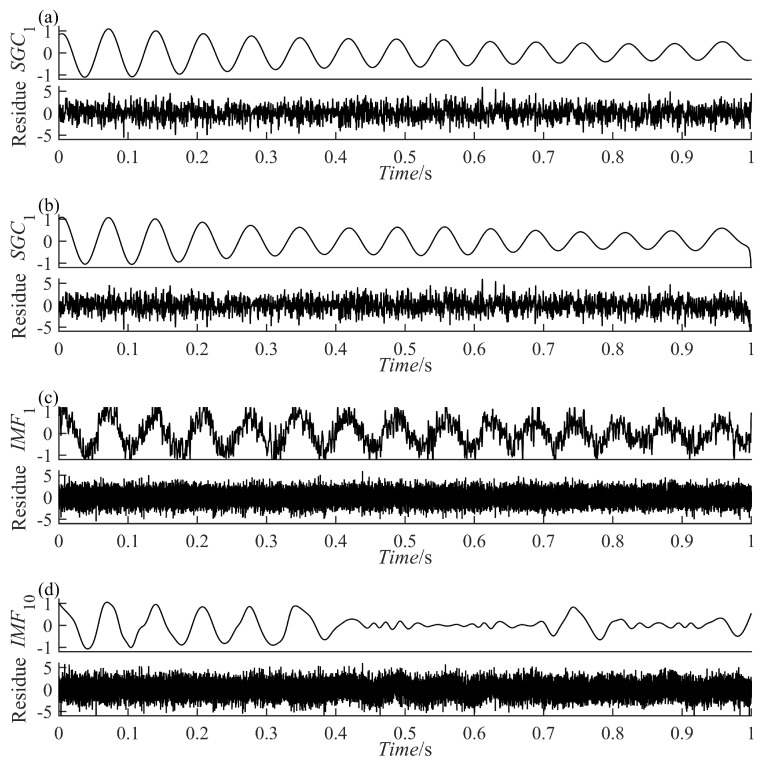
The decomposition results of the simulation signal y(t) at SNR = −10. (**a**) The PRSGMD decomposition result; (**b**) the SGMD decomposition result; (**c**) the VMD decomposition result; (**d**) the EEMD decomposition result.

**Figure 9:**
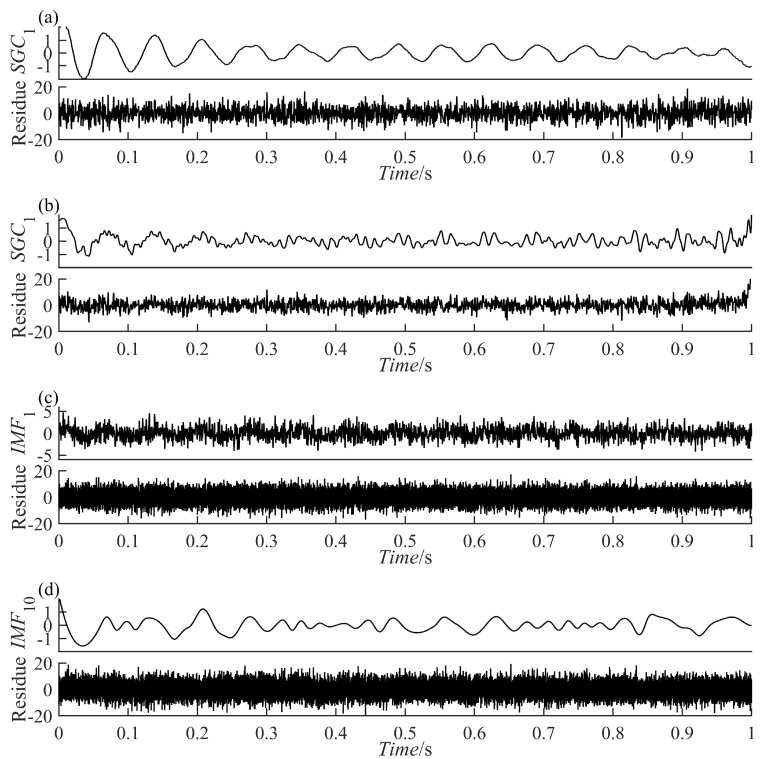
The decomposition results of the simulation signal y(t) at SNR = −20. (**a**) The PRSGMD decomposition result; (**b**) the SGMD decomposition result; (**c**) the VMD decomposition result; (**d**) the EEMD decomposition result.

The results of the signal y(t) decomposition by the PRSGMD, SGMD, VMD, and EEMD are depicted in Figure 7, Figure 8 and Figure 9 accordingly. Figure (a), (b), (c), and (d) are the PRSGMD, SGMD, VMD, and EEMD decomposition results of thesimulation signals, respectively. At the same time, to quantify the resistance to noise, the corresponding evaluation metrics are shown in Table 2. As can be seen from Figure 7, Figure 8 and Figure 9, when the signal-to-noise ratio is 5 dB, that is, when the noise is relatively weak, the four signal decomposition methods can effectively distinguish the vibration attenuation signal component from the noise, achieving the effect of signal and noise separation. Although the waveform of the effective component ${IMF}_{1}$ from the VMD contains burr and is not smooth enough, it is basically consistent with the waveform trend of the vibration attenuation signal in the simulation signal.

In terms of the accuracy of decomposition, according to the corresponding correlation coefficients in Table 2, it was concluded that the decomposition results of the SGMD, VMD, and EEMD are superior to PRSGMD, which is also reflected in the waveform of residual errors. The residuals of the VMD and EEMD are more consistent with the added noise from the perspective of time-domain averaging. However, the signal and noise separation of the PRSGMD is not thorough enough, so the waveform trend of the residual component contains weak AM characteristics. With the Gaussian white noise being strengthened, noise disturbance decreases the effectiveness and accuracy rate of the decomposition methods to varying degrees. When the signal-to-noise ratio is −10 dB, although the waveform of ${IMF}_{1}$ component of the VMD still shows a general trend of oscillation, the sawtooth fluctuation in the entire time domain seriously inhibits the attenuation characteristics of the component. At the same time, the curve of the ${\;IMF}_{10}$ component of EEMD has obvious waveform loss. The effective components of the PRSGMD and SGMD still maintain relatively high waveform similarity, and the corresponding correlation coefficient and energy error index also provide data support from the side. Although the accuracy of decomposition is slightly reduced, the effectiveness of decomposition is guaranteed, and the signal and noise separation are realized. When the SNR is −20 dB, the amplitude fluctuation of ${IMF}_{1}$ component of the VMD obviously exceeds the amplitude limit of $y_{1}\left(t\right)$ of the raw signal, and signal-noise aliasing is serious. The amplitude of the EEMD’s ${IMF}_{10}$ component varies in acceptable bounds, but the waveform is clearly missing. The smooth property of ${SGC}_{1}$ component of the SGMD is corrupted by noise; at the same time, its reliability declines with the end effect. In contrast, the ${SGC}_{1}$ component of the PRSGMD with slight distortion in some of the peaks and valleys and slightly diminished AM/FM characteristics still retains the waveform of the raw signal $y_{1}\left(t\right)$, generally. Its r1 (correlation coefficient) index is approximately 0.8, and the E1 (energy error) is the minimum among all methods. This method still achieves effective decomposition results despite the disturbance of intense noise.

The results of the above simulation analysis show that although the accuracy of the PRSGMD method is slightly lower than the other three methods when the noise intensity is weak, the effectiveness of the VMD and EEMD methods is significantly reduced or even ineffective when the noise intensity is increased. Although the SGMD has specific anti-noise performance, the decomposition performance cannot meet the requirements in the environment of high-intensity noise. The PRSGMD method is suitable for separating signal and noise, achieves a superior decomposition effect under the noise disturbance of different signal-to-noise ratios, and has favorable anti-noise performance. In terms of computational efficiency, although the decomposition time of the PRSGMD method is less than that of the SGMD method, the decomposition time is still higher than that of the VMD and EEMD methods. There is a need to optimize the filter parameters in the PRSGMD method to reduce decomposition time and improve decomposition efficiency.

## 4. The Application of the PRSGMD Method in the Diagnosis of Rolling Bearing Faults

**Figure 10:**
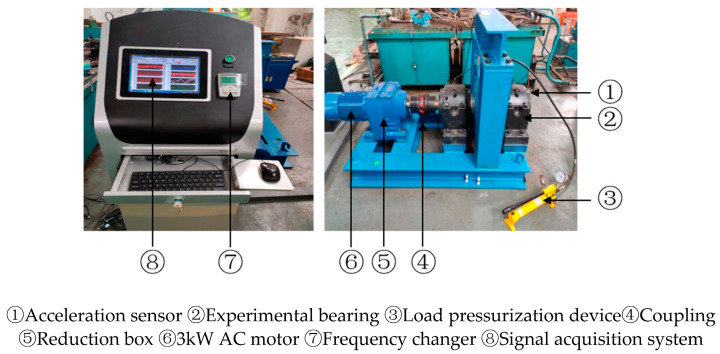
Experimental bench for hub bearing failures in variable speed overhead cranes.

To further explain the excellencies and practicality of the PRSGMD method, it was applied to rolling bearing vibration signals analysis. The bearing failure experimental equipment is given in Figure 10. The main constituent parts of the experimental equipment are the AC motor, frequency changer, gearbox, support frame, rotation shaft, coupling, acceleration sensor, load pressurization device, experimental bearing, acquisition card, VK702 signal acquisition system, etc. The bearings used in the experimental equipment are SKF 22238-MB spherical roller bearings. Before the experiment, the cage fault was set using the EDM wire-cutting machining technique, and the acceleration sensor was installed in the motor drive end housing. The frequency of sampling of the vibration signals was fixed at 1000 Hz, and each data sample selected in this paper contains 20,000 data points. The speed set for experiments was 40 rpm.

Using the Envelope Spectrumes (ES) analysis method to diagnose all the data in this dataset, the data were categorized as diagnosable (Y), with an ambiguous diagnosis (A), and completely undiagnosable (U). Therefore, a new signal analysis method can be applied to data that are completely undiagnosable (U) if it is necessary to test their practicality for rolling bearing fault diagnosis. This method can be applicationed to data that are completely undiagnosticable (U) if a good diagnostic result is achieved. The parameters associated with the data are given in Table 3. The rotation frequency was calculated to be 0.67 Hz, and the fundamental train frequency (FTF) was 0.288 Hz.

**Figure 11:**
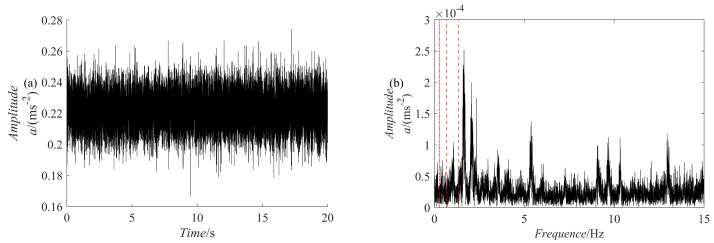
The time-domain diagram of the vibration signal and its ES. (**a**) The raw signal; (**b**) the raw signal’s ES.

**Figure 12:**
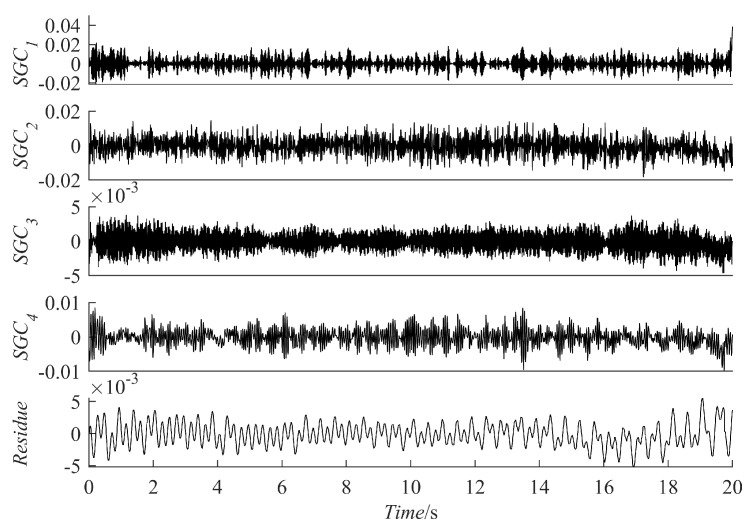
Results of vibration signal decomposed using PRSGMD.

**Figure 13:**
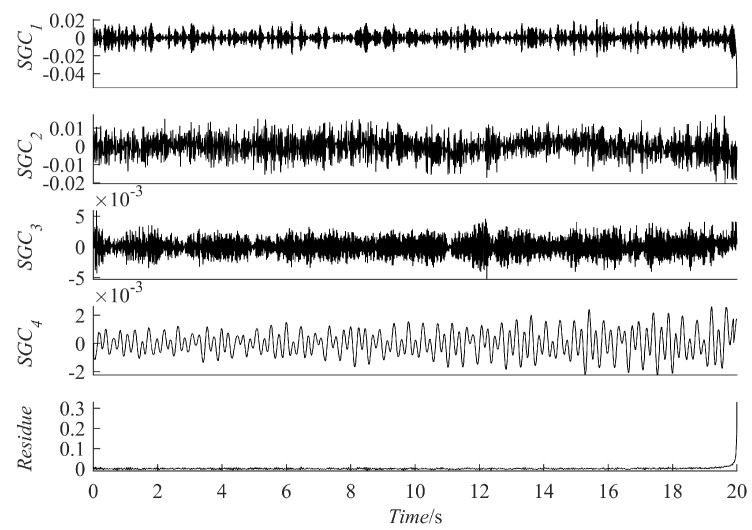
Results of vibration signal decomposed using SGMD.

**Figure 14:**
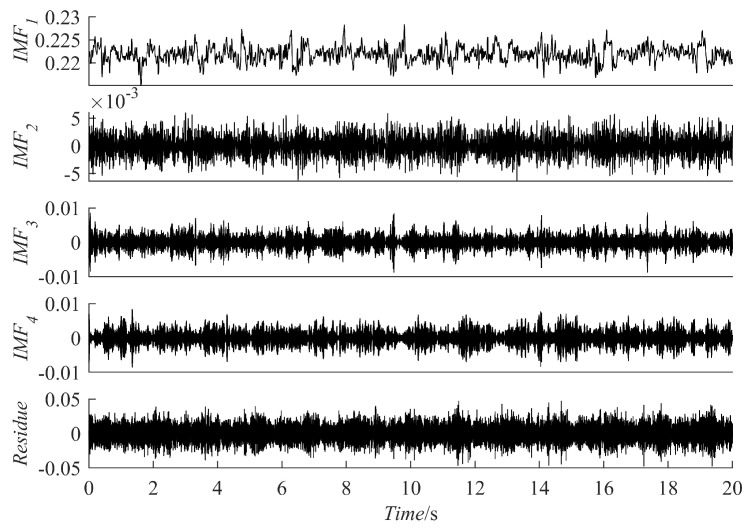
Results of vibration signal decomposed using VMD.

**Figure 15:**
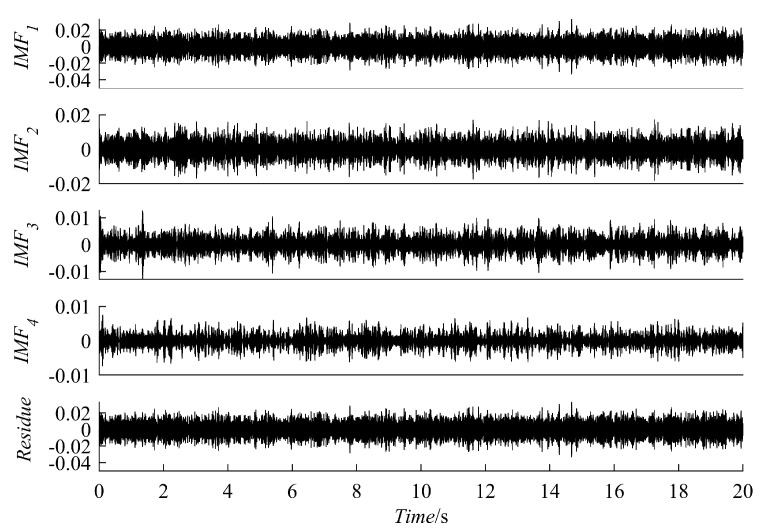
Results of vibration signal decomposed using EEMD.

**Figure 16:**
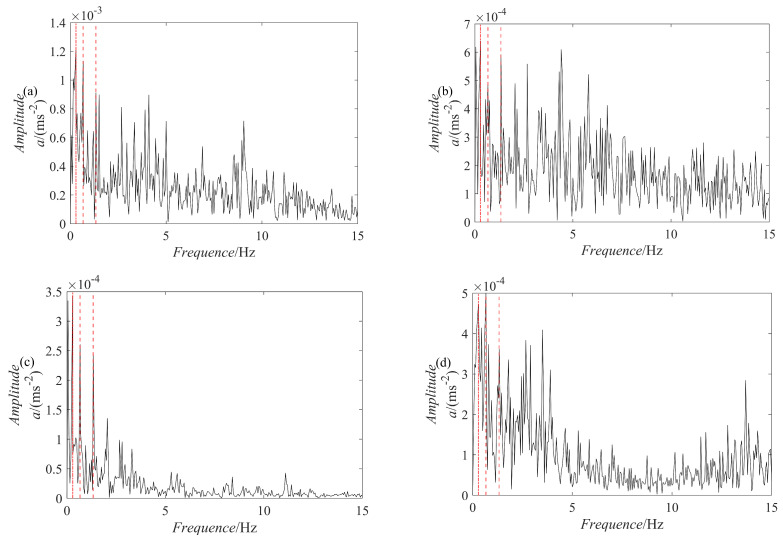
The ES of the previous four components decomposed using the PRSGMD method. (**a**–**d**) The ES of *SGC*_1_, *SGC*_2_, *SGC*_3_, and *SGC*_4,_ respectively.

**Figure 17:**
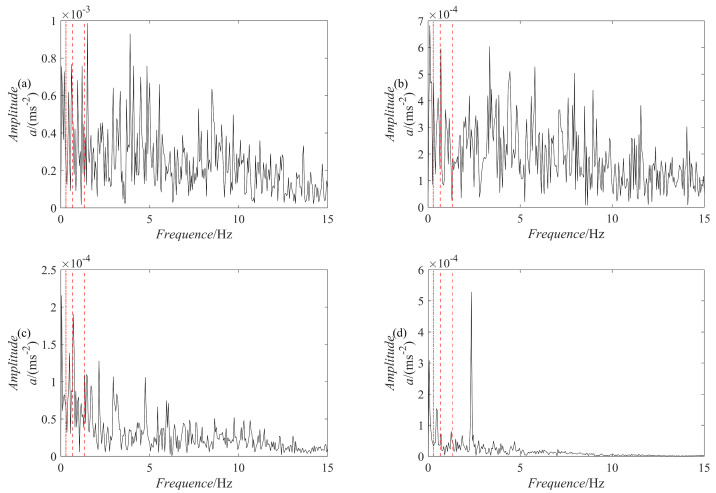
The ES of the previous four components decomposed using the SGMD method. (**a**–**d**) The ES of *SGC*_1_, *SGC*_2_, *SGC*_3_, and *SGC*_4_, respectively.

**Figure 18:**
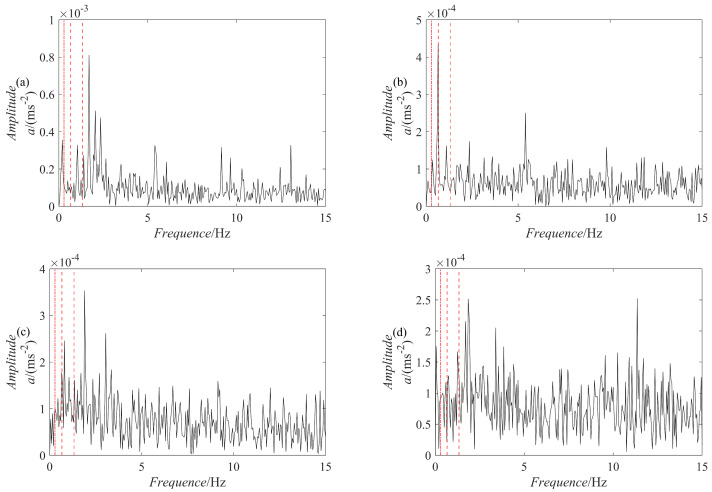
The ES of the previous four components decomposed using the VMD method. (**a**–**d**) The ES of *IMF*_1_, *IMF*_2_, *IMF*_3_, and *IMF*_4_, respectively.

**Figure 19:**
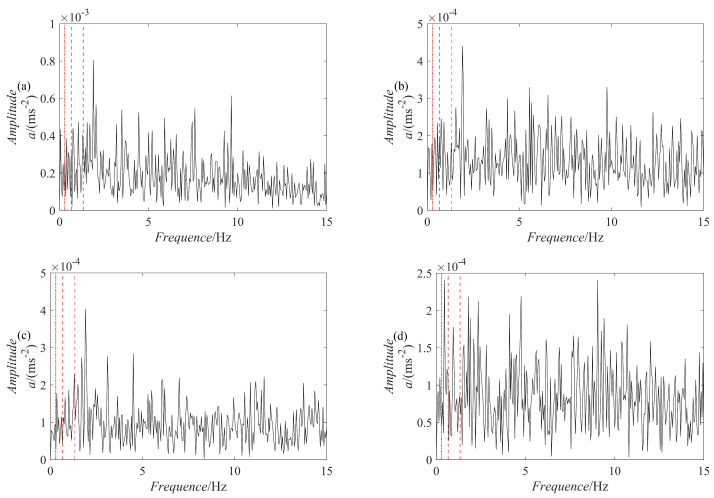
The ES of the previous four components decomposed using the EEMD method. (**a**–**d**) The ES of *IMF*_1_, *IMF*_2_, *IMF*_3_, and *IMF*_4_, respectively.

For comparison, five methods are used for fault diagnosis: ES analysis, EEMD decomposition followed by ES analysis, VMD decomposition followed by ES analysis, SGMD decomposition of the raw signal followed by ES analysis, and PRSGMD decomposition followed by ES analysis. Figure 11 presents the raw signal together with its ES. Figure 12, Figure 13, Figure 14, Figure 15, Figure 16, Figure 17, Figure 18 and Figure 19 present the results using four various decomposition methods together with their ES. A more apparent impulse response sequence was obtained using the PRSGMD method, as shown from SGC_4_ in Figure 12. Figure 11b’s dashed line shows the harmonics of $f_{r}$ and Figure 11b’s dash-dotted line shows the harmonics of FTF. Furthermore, Figure 11b’ES also indicates that the rotation frequency and fault information are masked.

The ES of the PRSGMD decomposition results in Figure 16 can clearly distinguish the harmonics of the rotation frequency ($f_{r}$, ${2\;\times \;f}_{r}$) and the first harmonic of the FTF. The ES of the SGMD and VMD decomposition results in Figure 17 and Figure 18 do not have obvious FTF harmonics, but the first harmonic of the rotation frequency can be clearly distinguished in Figure 17b,c and Figure 18b. However, inthe ES of the EEMD decomposition results in Figure 19, it is difficult to distinguish the obvious FTF harmonics. As a result, the PRSGMD method effectively diagnoses cage faults. According to the above classification of diagnostic effects, the diagnosis effects of the five methods of ES analysis, the EEMD decomposition followed by ES analysis, the VMD decomposition followed by ES analysis, the SGMD decomposition followed by ES analysis, and the PRSGMD decomposition followed by ES analysis were classified as U, U, U, U, and Y, respectively.

The decomposition times of the raw vibration signal using the four decomposition methods are recorded in Table 4. During the decomposition of the actual vibration signal, the PRSGMD method has a shorter decomposition time compared to the SGMD method, which improves the efficiency of the decomposition of the raw vibration signal, but the decomposition time is still higher than that of the VMD and EEMD methods.

From the above analysis results, the PRSGMD method can effectively diagnose the faults of rolling bearings. Moreover, compared with the direct ES of the raw signal or using EEMD, VMD, and SGMD methods to decompose, followed by the ES, the PRSGMD method can more accurately extract the fault characteristics of rolling bearings from noise signals.

## 5. Conclusions

Taking aim at SGMD suffersing from a rapid decrease in calculation efficiency as the amount of data increases and the impact of the invalid SGC on the decomposition accuracy during reconstruction, in this paper, the PRSGMD method is proposed for the extraction of rolling bearing fault characteristics information, with the following specific conclusions:

1. The RCMFE operator is constructed to evaluate the validity of each initial single component after reconstruction, and the physically meaningful SGC components can be effectively obtained by constraining the residual energy to be minimal.
2. Compared with the raw SGMD method, PRSGMD only needs to process partthe s of the initial single components that contain significant modes and does not reduce the operational efficiency as the amount of data increases, which can effectively improve the decomposition speed.
3. Simulation and experimental analysis results demonstrate that relative to SGMD, EEMD, and VMD, PRSGMD is advantageous in suppressing endpoint effects and modal confusion, resistance to noise property, and increasing the orthogonality and accuracy of the components, while the overall time consumption of PRSGMD is lower than that of SGMD.

It is worth mentioning that the processing of rolling bearing vibration data for variable operating conditions and the convergence of the algorithm in PRSGMD need further exploration.

## Figures and Tables

**Table 1:** Evaluation metrics of the comprehensive performance of the PRSGMD, SGMD, VMD, and EEMD.

Method	r1	r2	E1	E2	IO	T (s)
PRSGMD	0.9929	0.9947	0.0142	0.0106	0.0300	3.9463
SGMD	0.9913	0.9933	0.0183	0.0136	0.0819	8.0946
VMD	0.9394	0.9736	0.1240	1.0180	0.2222	1.7803
EEMD	0.9151	08812	0.1627	3.8468	0.0823	0.1201

**Table 2:** Evaluation metrics of the components of y(t) obtained using PRSGMD, SGMD, VMD, and EEMD.

Method	SNR	r1	E1	T (s)
PRSGMD	5	0.9868	0.0282	1.385
	−10	0.9205	0.1506	2.986
	−20	0.7968	0.3675	4.746
SGMD	5	0.9769	0.0349	10.37
	−10	0.8643	0.2341	12.94
	−20	0.5488	1.1281	10.46
VMD	5	0.9646	0.0448	4.948
	−10	0.8223	0.3410	6.964
	−20	0.3122	1.7078	7.420
EEMD	5	0.9589	0.0636	0.3176
	−10	0.7603	0.5014	0.3708
	−20	0.6054	0.7940	0.4676

**Table 3:** Parameters related to vibration signals.

Type	Type of Failure	Sample Frequency	Bearing Speed	Frequency of Rotation
SKF 22238-MB	Cage failure	1 kHz	40 rpm	0.67 Hz

**Table 4:** Decomposition time for the four decomposition methods.

Decomposition Methods	PRSGMD	SGMD	VMD	EEMD
T(s)	6.9636	42.4933	2.2539	1.1779

## Data Availability

Not applicable.
